# EM Algorithm for Estimating the Parameters of Weibull Competing Risk Model

**DOI:** 10.1155/2021/1179856

**Published:** 2021-10-21

**Authors:** Mohamed Kayid

**Affiliations:** Department of Statistics and Operations Research, College of Science, King Saud University, Riyadh, Saudi Arabia

## Abstract

One of the most commonly used models in survival analysis is the additive Weibull model and its generalizations. They are well suited for modeling bathtub-shaped hazard rates that are a natural form of the hazard rate. Although they have some advantages, the maximum likelihood and the least square estimators are biased and have poor performance when the data set contains a large number of parameters. As an alternative, the expectation-maximization (EM) algorithm was applied to estimate the parameters of the additive Weibull model. The accuracy of the parameter estimates and the simulation study confirmed the advantages of the EM algorithm.

## 1. Introduction

There are many situations in which the hazard rate function shows a bathtub shape (BTS) with three life periods, early decreasing hazard rate period, useful period (where the hazard rate is approximately constant), and eventually increasing hazard rate period. Lai et al. [1] and Nadarajah [2] presented a list of distributions with BTS hazard rate. Many authors assumed a BTS hazard rate model in their research, and among them, we can point to Block et al. [3], Glaser [4], Leemis and Beneke [5], Mi [6, 7], Mitra and Basu [8], and Noughabi et al. [9].

Xie and Lai [10] and Jiang and Murthy [11, 12] studied some aspects of the additive Weibull model with the hazard rate function:

(1) $$\begin{array}{c} h\left(x\right)=\alpha_{1}\lambda_{1}^{\alpha_{1}}x^{\alpha_{1}-1}+\alpha_{2}\lambda_{2}^{\alpha_{2}}x^{\alpha_{2}-1},x\geq 0, \end{array}$$

as a good candidate for describing the bathtub-shaped failure rate function. They urged that the statistical inference about this model is complex due to the number of the parameters, and as a remedy, they suggested the reduced model by considering *λ*_1_ = *λ*_2_ and *α*_2_ = 1/*α*_1_ which still accommodates the bathtub-shaped hazard rate.

Lai et al. [13] added one constant magnitude to the additive Weibull hazard rate (1) to provide more realistic model:

(2) $$\begin{array}{c} h\left(x\right)=\alpha_{1}\lambda_{1}^{\alpha_{1}}x^{\alpha_{1}-1}+\alpha_{2}\lambda_{2}^{\alpha_{2}}x^{\alpha_{2}-1}+\lambda_{3},x\geq 0. \end{array}$$

In addition, Bebbington et al. [14] considered the additive Weibull models (1) and (2) in their research to express the concept of the useful period of life of a bathtub-shaped hazard rate distribution. They concluded that the additive Weibull model is sufficiently flexible to describe a bathtub-shaped hazard rate. The common estimator of the parameters of these models is the maximum likelihood estimator (MLE).

The EM algorithm is an iterative algorithm for estimating parameters of models involving latent variables, e.g., when data is derived from a mixture or competing risk model. It was used by Dempster et al. [15], Balakrishnan et al. [16], Davies et al. [17], Yang et al. [18], and Okamura and Dohi [19] to estimate the parameters in their models. In this paper, we use the EM algorithm to estimate the parameters of the additive model (2) and show by a simulation study that this algorithm gives a better estimate than the MLE and the least square estimator (LSE).

The paper has been organized as the following. In Section 2, we present a short representation of the MLE and LSE of the parameters. Then, the EM algorithm has been discussed.  Section 3 provides a simulation study for comparing the results related to MLE, LSE, and EM estimator. In Section 4, a data set has been analyzed to show the applicability of the proposed estimators.

## 2. The MLE, Least Square, and EM

Let *x*_1_, *x*_2_, ⋯, *x*_*n*_ represents a realization of an iid random sample of the additive Weibull hazard rate distribution with the hazard rate (2). The log-likelihood function has five parameters and is of the form

(3) $$\begin{array}{c} l\left(\alpha_{1},\lambda_{1},\alpha_{2},\lambda_{2},\lambda_{3}\right)=\overset{n}{\underset{i=1}{\sum }}\ln\left(\alpha_{1}\lambda_{1}^{\alpha_{1}}x_{i}^{\alpha_{1}-1}+\alpha_{2}\lambda_{2}^{\alpha_{2}}x_{i}^{\alpha_{2}-1}+\lambda_{3}\right)-\overset{n}{\underset{i=1}{\sum }}\left({\left(\lambda_{1}x_{i}\right)}^{\alpha_{1}}+{\left(\lambda_{2}x_{i}\right)}^{\alpha_{2}}+\lambda_{3}x_{i}\right). \end{array}$$

To find the MLE, we should find admissible values of (*α*_1_, *λ*_1_, *α*_2_, *λ*_2_, *λ*_3_) which maximize the log-likelihood function.

To find the LSE of the parameters, we apply the reliability function related to the hazard rate model (2) which is

(4) $$\begin{array}{c} R\left(x\right)=e^{-{\left(\lambda_{1}x\right)}_{1}^{\alpha }-{\left(\lambda_{2}x\right)}_{2}^{\alpha }-\lambda_{3}x},x\geq 0. \end{array}$$

The empirical reliability function is defined to be

(5) $$\begin{array}{c} \tilde{R}\left(t\right)=\frac{1}{n}\overset{n}{\underset{i=1}{\sum }}I\left(t<x_{i}\right), \end{array}$$

in which the indicator function *I*(*t* < *x*_*i*_) equals 1 when *t* < *x*_*i*_ and otherwise is 0. So, the LSE of the parameters can be computed by minimizing the following sum of squares of errors in terms of (*α*_1_, *λ*_1_, *α*_2_, *λ*_2_, *λ*_3_).

(6) $$\begin{array}{c} S^{2}=\overset{n}{\underset{i=1}{\sum }}{\left(\tilde{R}\left(x_{i}\right)-e^{-{\left(\lambda_{1}x_{i}\right)}^{\alpha_{1}}-{\left(\lambda_{2}x_{i}\right)}^{\alpha_{2}}-\lambda_{3}x_{i}}\right)}^{2}. \end{array}$$

### 2.1. The EM Algorithm

Let *X*_1*i*_, *X*_2*i*_, and *X*_3*i*_, *i* = 1, 2, ⋯, *n* follows from the Weibull distributions with parameters (*α*_1_, *λ*_1_) and (*α*_2_, *λ*_2_) and the exponential distribution with mean 1/*λ*_3_, respectively. Assume that in a lifetime experiment, the observations are realizations of the competing risk random variable *X*_*i*_ = min{*X*_1*i*_, *X*_2*i*_, *X*_3*i*_}. This means that the lifetime event may be due to one of three competing causes. Let the latent random variable *Z*_*i*_ with the support {1, 2, 3} such that

(7) $$\begin{array}{c} Z_{i}=\left(\begin{array}{cc} 1 & \text{when}X_{1i}\leq \min\left\{X_{2i},X_{3i}\right\},\\ 2 & \text{when}X_{2i}\leq \min\left\{X_{1i},X_{3i}\right\},\\ 3 & \text{when}X_{3i}\leq \min\left\{X_{1i},X_{2i}\right\}. \end{array}\right. \end{array}$$

With these notations, the likelihood function is

(8) $$\begin{array}{c} L\left(\theta ;x,z\right)=\overset{n}{\underset{i=1}{\prod }}\left(\overset{3}{\underset{j=1}{\prod }}f{\left(Z_{i}=j\;|\;X_{i}=x_{i},\theta \right)}^{I\left(Z_{i}=j\right)}\right)f\left(X_{i}=x_{i}\;|\;\theta \right)=\overset{n}{\underset{i=1}{\prod }}\frac{\prod_{j=1}^{3}\lambda_{j}{\left(x_{i}\;|\;\theta \right)}^{I\left(Z_{i}=j\right)}}{\lambda_{1}\left(x_{i}\;|\;\theta \right)+\lambda_{2}\left(x_{i}\;|\;\theta \right)+\lambda_{3}\left(x_{i}\;|\;\theta \right)}\times \left(f_{1}\left(x_{i}\;|\;\theta \right)R_{2}\left(x_{i}\;|\;\theta \right)R_{3}\left(x_{i}\;|\;\theta \right)+f_{2}\left(x_{i}\;|\;\theta \right)R_{1}\left(x_{i}\;|\;\theta \right)R_{3}\left(x_{i}\;|\;\theta \right)\left.+f_{3}\left(x_{i}\;|\;\theta \right)R_{1}\left(x_{i}\;|\;\theta \right)R_{2}\left(x_{i}\;|\;\theta \right)\right)=\overset{n}{\underset{i=1}{\prod }}\left(\overset{3}{\underset{j=1}{\prod }}\lambda_{j}{\left(x_{i}\;|\;\theta \right)}^{I\left(Z_{i}=j\right)}\right)R_{1}\left(x_{i}\;|\;\theta \right)R_{2}\left(x_{i}\;|\;\theta \right)\left.R_{3}\left(x_{i}\;|\;\theta \right)\right),\right. \end{array}$$

where *R*_*j*_(*x* | *θ*), *f*_*j*_(*x* | *θ*), and *λ*_*j*_(*x* | *θ*) show the corresponding reliability function, the density function, and the hazard rate function of *X*_*ji*_, *j* = 1, 2, 3. Then, the log-likelihood function is

(9) $$\begin{array}{c} l\left(\theta ;x,z\right)=\ln L\left(\theta ;x,z\right)=\overset{n}{\underset{i=1}{\sum }}\overset{3}{\underset{j=1}{\sum }}I\left(Z_{i}=j\right)\ln\lambda_{j}\left(x_{i}\;|\;\theta \right)+\overset{n}{\underset{i=1}{\sum }}\overset{3}{\underset{j=1}{\sum }}\ln R_{j}\left(x_{i}\;|\;\theta \right). \end{array}$$

The EM algorithm is an iterative algorithm, and every iteration of it consists of two consecutive steps, namely, the E step and the M step. In the E step, the expectation of the log-likelihood with respect to the estimate of the conditional probabilities of the latent variables has been constructed. Then, in the M step, the constructed expectation of the E step is maximized to compute the estimate of the parameters in the current iteration.

### 2.2. The E Step

Suppose that the estimate of *θ* at iteration *t* be denoted by *θ*_*t*_, then the conditional distribution of *Z*_*i*_ is

(10) $$\begin{array}{c} p_{i1,t}=P\left(Z_{i}=1\;|\;X_{i}=x_{i},\theta_{t}\right)=\frac{f_{1}\left(x_{i}\;|\;\theta_{t}\right)R_{2}\left(x_{i}\;|\;\theta_{t}\right)R_{3}\left(x_{i}\;|\;\theta_{t}\right)}{f\left(x_{i}\;|\;\theta_{t}\right)}, \end{array}$$

(11) $$\begin{array}{c} p_{i2,t}=P\left(Z_{i}=2\;|\;X_{i}=x_{i},\theta_{t}\right)=\frac{f_{2}\left(x_{i}\;|\;\theta_{t}\right)R_{1}\left(x_{i}\;|\;\theta_{t}\right)R_{3}\left(x_{i}\;|\;\theta_{t}\right)}{f\left(x_{i}\;|\;\theta_{t}\right)}, \end{array}$$

and *p*_*i*3,*t*_ = 1 − *p*_*i*1,*t*_ − *p*_*i*2,*t*_ where

(12) $$\begin{array}{c} f\left(x_{i}\;|\;\theta_{t}\right)=f_{1}\left(x_{i}\;|\;\theta_{t}\right)R_{2}\left(x_{i}\;|\;\theta_{t}\right)R_{3}\left(x_{i}\;|\;\theta_{t}\right)+f_{2}\left(x_{i}\;|\;\theta_{t}\right)R_{1}\left(x_{i}\;|\;\theta_{t}\right)R_{3}\left(x_{i}\;|\;\theta_{t}\right)+f_{3}\left(x_{i}\;|\;\theta_{t}\right)R_{1}\left(x_{i}\;|\;\theta_{t}\right)R_{2}\left(x_{i}\;|\;\theta_{t}\right). \end{array}$$

The probabilities *p*_*i*1,*t*_, *p*_*i*2,*t*_, and *p*_*i*3,*t*_ are called the membership probabilities.

Now we define the expectation of the log-likelihood function (8) with respect to the conditional distribution of *Z*_*i*_.

(13) $$\begin{array}{c} Q\left(\theta \;|\;\theta_{t}\right)=E_{Z\;|\;X,\theta_{t}}\left(l\left(\theta ;x,Z\right)\right)=\overset{\text{n}}{\underset{i=1}{\sum }}p_{1i,t}\left(\ln\left(\alpha_{1}\lambda_{1}^{\alpha_{1}}x_{i}^{\alpha_{1}-1}\right)-{\left(\lambda_{1}x_{i}\right)}^{\alpha_{1}}-{\left(\lambda_{2}x_{i}\right)}^{\alpha_{2}}-\lambda_{3}x_{i}\right)+\overset{n}{\underset{i=1}{\sum }}p_{2i,t}\left(\ln\left(\alpha_{2}\lambda_{2}^{\alpha_{2}}x_{i}^{\alpha_{2}-1}\right)-{\left(\lambda_{1}x_{i}\right)}^{\alpha_{1}}-{\left(\lambda_{2}x_{i}\right)}^{\alpha_{2}}-\lambda_{3}x_{i}\right)+\overset{n}{\underset{i=1}{\sum }}p_{3i,t}\left(\ln\lambda_{3}-{\left(\lambda_{1}x_{i}\right)}^{\alpha_{1}}-{\left(\lambda_{2}x_{i}\right)}^{\alpha_{2}}-\lambda_{3}x_{i}\right)=\overset{n}{\underset{i=1}{\sum }}p_{1i,t}\ln\alpha_{1}\lambda_{1}^{\alpha_{1}}x_{i}^{\alpha_{1}-1}-\overset{n}{\underset{i=1}{\sum }}{\left(\lambda_{1}x_{i}\right)}^{\alpha_{1}}+\overset{n}{\underset{i=1}{\sum }}p_{2i,t}\ln\alpha_{2}\lambda_{2}^{\alpha_{2}}x_{i}^{\alpha_{2}-1}-\overset{n}{\underset{i=1}{\sum }}{\left(\lambda_{2}x_{i}\right)}^{\alpha_{2}}+\overset{n}{\underset{i=1}{\sum }}p_{3i,t}\ln\lambda_{3}-\overset{n}{\underset{i=1}{\sum }}\lambda_{3}x_{i}. \end{array}$$

So the *Q*(*θ* | *θ*_*t*_) can be written as sum of three distinct expressions *Q*_1_(*θ* | *θ*_*t*_), *Q*_2_(*θ* | *θ*_*t*_), and *Q*_3_(*θ* | *θ*_*t*_), where

(14) $$\begin{array}{c} Q_{1}\left(\theta \;|\;\theta_{t}\right)\overset{n}{\underset{i=1}{\sum }}p_{1i,t}\ln\alpha_{1}\lambda_{1}^{\alpha_{1}}x_{i}^{\alpha_{1}-1}-\overset{n}{\underset{i=1}{\sum }}{\left(\lambda_{1}x_{i}\right)}^{\alpha_{1}}, \end{array}$$

(15) $$\begin{array}{c} Q_{2}\left(\theta \;|\;\theta_{t}\right)=\overset{n}{\underset{i=1}{\sum }}p_{2i,t}\ln\alpha_{2}\lambda_{2}^{\alpha_{2}}x_{i}^{\alpha_{2}-1}-\overset{n}{\underset{i=1}{\sum }}{\left(\lambda_{2}x_{i}\right)}^{\alpha_{2}}, \end{array}$$

(16) $$\begin{array}{c} Q_{3}\left(\theta \;|\;\theta_{t}\right)=\overset{n}{\underset{i=1}{\sum }}p_{3i,t}\ln\lambda_{3}-\overset{n}{\underset{i=1}{\sum }}\lambda_{3}x_{i}. \end{array}$$

These statements will be applied in the M step, to compute the estimates of the parameters.

### 2.3. The M Step

The estimate of the parameters at iteration *t* + 1 can be obtained by

(17) $$\begin{array}{c} {\hat{\alpha }}_{1,t+1},{\hat{\lambda }}_{1,t+1}=\arg\underset{\alpha ,\lambda }{\max}Q_{1}\left(\theta \;|\;\theta_{t}\right), \end{array}$$

(18) $$\begin{array}{c} {\hat{\alpha }}_{2,t+1},{\hat{\lambda }}_{2,t+1}=\arg\underset{\alpha ,\lambda }{\max}Q_{2}\left(\theta \;|\;\theta_{t}\right), \end{array}$$

(19) $$\begin{array}{c} {\hat{\lambda }}_{3,t+1}=\arg\underset{\lambda }{\max}Q_{3}\left(\theta \;|\;\theta_{t}\right). \end{array}$$

We should optimize *Q*_1_(*θ* | *θ*_*t*_) and *Q*_2_(*θ* | *θ*_*t*_) numerically since they have not closed form for their critical points. But, the point which maximizes *Q*_3_(*θ* | *θ*_*t*_) has a closed form, and by solving the equation (*∂*/*∂λ*_3_)*Q*_3_ = 0, we have

(20) $$\begin{array}{c} {\hat{\lambda }}_{3,t+1}=\frac{\sum_{i=1}^{n}p_{3i,t}}{\sum_{i=1}^{n}x_{i}}. \end{array}$$

The iterative process can be concluded if *Q*(*θ*_*t*+1_ | *θ*_*t*+1_) < *Q*(*θ*_*t*_ | *θ*_*t*_) + *ε* for some small predefined *ε*.

## 3. Simulation Study

To provide a random instance of the competing risk model with hazard rate (2), we simulate one random instance of Weibull with parameters (*α*_1_, *λ*_1_), namely, *X*_11_, one random instance of Weibull with parameters (*α*_2_, *λ*_2_), namely, *X*_12_, and one random instance of the exponential distribution with parameter *λ*_3_, namely, *X*_13_. Then, the random variable *X*_1_ = min{*X*_11_, *X*_12_, *X*_13_} follows from the desired competing risk model.

In every run of the simulation study, we drive *r* = 500 replicates of samples of sizes *n* = 50 and 100. Then, for each sample, the parameters have been estimated applying the MLE, LSE, or EM algorithm (see Supplementary Materials for all R codes used for simulation study). Every cell of Table 1 shows the results of one run. The results contain the bias (*B*), the absolute bias (AB), and the mean squared error (MSE) which, for example for *α*_1_, have been computed by the following relations.

(21) $$\begin{array}{c} B_{\alpha_{1}}=\frac{1}{r}\overset{r}{\underset{i=1}{\sum }}\left({\hat{\alpha }}_{1i}-\alpha_{1}\right), \end{array}$$

(22) $$\begin{array}{c} \text{A}{\text{B}}_{\alpha_{1}}=\frac{1}{r}\overset{r}{\underset{i=1}{\sum }}\left|{\hat{\alpha }}_{1i}-\alpha_{1}\right|, \end{array}$$

(23) $$\begin{array}{c} \text{MS}{\text{E}}_{\alpha_{1}}=\frac{1}{r}\overset{r}{\underset{i=1}{\sum }}{\left({\overset{\wedge }{\alpha }}_{1i}-\alpha_{1}\right)}^{2}, \end{array}$$

where ${\hat{\alpha }}_{1i}$ is the estimate of *α*_1_ based on the *i*th replication. Some important observations of the simulation results have been pointed out in the following.

- As sample size increases, the AB and MSE decrease in all approaches
- The EM estimator outperforms the LSE and MLE in terms of AB and MSE in all cases
- The LSE outperforms the MLE in terms of AB and MSE in all cases

## 4. Applications

**Figure 1:**
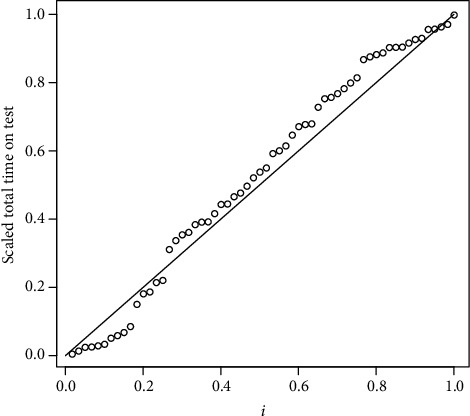
The scaled TTT transform plot for data sets of Table 2.

**Figure 2:**
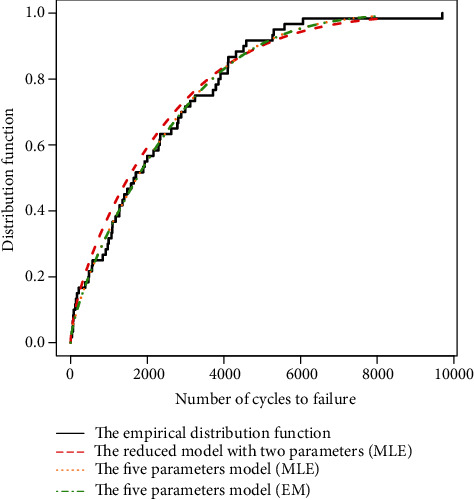
The empirical CDF and some fitted models to data set of Table 2.

Lawless [20] analyzed failure times of some electrical appliances. The scaled TTT transform plot drawn in Figure 1 shows a bathtub shape for the hazard rate function. This gives us some nonparametric information indicating that the data come from a BT hazard rate model. So we tried to fit some distributions accommodating bathtub-shaped hazard rate to this data set. We use the MLE and the EM algorithm to fit the five parameters competing risk model (2). Also, the reduced model with the reliability function

(24) $$\begin{array}{c} R\left(x\right)=\exp\left\{-{\left(\lambda x\right)}^{\alpha }-{\left(\lambda x\right)}^{1/\alpha }\right\}, \end{array}$$

has been fitted by computing the MLE of the parameters. In Figure 2, the cumulative distribution function (CDF) for fitted models has been drawn. There is a significant distance between the fitted model (14) and the empirical CDF which shows that the reduced model may be improper in some examples.

**Figure 3:**
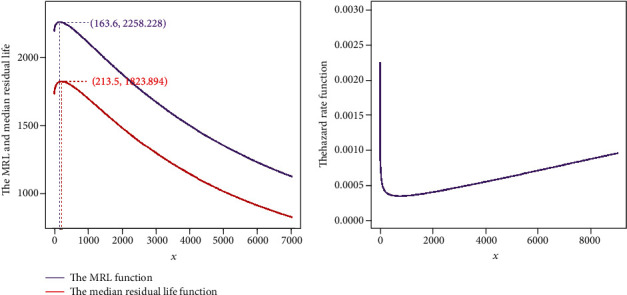
(a) The mean residual life and the median residual life of the fitted model. (b) The hazard rate function of this fitted model.

Moreover, some results of fit have been abstracted in Table 3. Based on the Kolmogorov-Smirnov (K-S) statistics and *p* value, the competing risk model (2) which has been fitted by the EM algorithm gives the best description of the data. The MLE has also provided good results, but it is worthy to denote that we applied the estimates of the EM algorithm as the initial values in the likelihood maximization process. All of the fitted models confirm a BT hazard rate model which was firstly recognized by the TTT transform plot. So it may be interesting to investigate the point which maximizes the mean residual life and/or the median residual life functions. These points are referred to burn-in points and show the time at which the component is in its most reliable condition. The left side of Figure 3 draws the mean residual life function along with the median residual life function related to the best fitted model. Also, the burn-in points related to both functions have been determined in the figure. The right side of Figure 2 draws the hazard rate function of this model and shows a BT hazard rate model.

## 5. Conclusion

The competing risk model of the baseline distribution Weibull plays a vital role in describing nonmonotone hazard rate models. One drawback of this model is that it has large number of the parameters which causes the estimation problem harder. Some authors suggested reduced versions to overcome this problem. But, there are many examples showing that the reduced model may not be proper. So, we implemented the EM algorithm for estimating the parameters. The simulation results confirm that this algorithm is better than MLE and LSE. As future works, such EM algorithm may be constructed for similar competing risk models or mixture models, for example, the gamma competing risk model with the following reliability function may be a good candidate:

(25) $$\begin{array}{c} R\left(x\right)=\frac{1}{\Gamma \left(\alpha_{1}\right)\Gamma \left(\alpha_{2}\right)}\Gamma \left(\alpha_{1},\lambda_{1}x\right)\Gamma \left(\alpha_{2},\lambda_{2}x\right),x\geq 0, \end{array}$$

in which Γ(*α*, *t*) = ∫_*t*_^∞^*y*^*α*−1^*e*^−*y*^*dy* is the upper incomplete gamma function and *α*_1_ > 0, *λ*_1_ > 0, *α*_2_ > 0, and *λ*_2_ > 0.

## Figures and Tables

**Table 1:** Every cell consists of the bias, the absolute bias, and the mean squared error for five parameters *α*_1_, *λ*_1_, *α*_2_, *λ*_2_, and *λ*_3_ from top to bottom, respectively.

	*n*	The EM algorithm			The LSE			The MLE		
		B	AB	MSE	B	AB	MSE	B	AB	MSE
*α*_1_ = 0.8 *λ*_1_ = 0.01 *α*_2_ = 1.3 *λ*_2_ = 0.02 *λ*_3_ = 0.04	50	0.114788	0.209461	0.073327	0.064984	0.266447	0.295491	0.150792	0.278228	0.475591
		-0.001410	0.003386	0.000017	0.015999	0.020659	0.000707	0.026929	0.030571	0.001399
		0.354883	0.414357	0.325645	0.450476	0.596059	1.216075	1.710262	1.829587	30.45458
		0.001644	0.004211	0.000030	0.0008324	0.015219	0.000326	0.003705	0.013934	0.000315
		0.006223	0.007680	0.000101	-0.018570	0.025329	0.000851	-0.025577	0.031822	0.001172
	100	0.090420	0.161060	0.041728	0.075634	0.208668	0.090426	0.074398	0.221743	0.092066
		-0.001857	0.002883	0.000011	0.014107	0.018573	0.000558	0.022671	0.027087	0.001132
		0.269857	0.300074	0.151175	0.351361	0.551910	0.787756	1.266889	1.407221	38.77269
		0.000711	0.002802	0.000012	0.007800	0.013041	0.000264	0.000171	0.012493	0.000243
		0.006117	0.006801	0.000072	-0.017343	0.024008	0.000788	-0.018373	0.027122	0.000936

*α*_1_ = 1.1 *λ*_1_ = 0.1 *α*_2_ = 0.9 *λ*_2_ = 0.2 *λ*_3_ = 0.3	50	0.087247	0.189355	0.067184	0.388006	0.476704	0.656696	1.686055	1.736100	193.3874
		0.010944	0.026164	0.001247	0.131134	0.184852	0.053799	0.057770	0.124714	0.025303
		0.049117	0.165828	0.047906	-0.011756	0.306156	0.273852	0.019276	0.239581	0.241092
		0.011833	0.033445	0.001913	0.082536	0.215583	0.070223	0.161446	0.250880	0.084662
		0.010969	0.033325	0.001867	-0.149815	0.212298	0.056467	-0.165021	0.245053	0.068212
	100	0.057342	0.128094	0.027325	0.332300	0.424207	0.583780	0.919810	0.977701	8.022046
		0.005099	0.018084	0.000525	0.104450	0.160100	0.040268	0.030251	0.106644	0.017581
		0.035447	0.107349	0.018491	-0.011164	0.227723	0.132404	0.008467	0.179967	0.311876
		0.004282	0.023096	0.000883	0.087848	0.195858	0.057835	0.170213	0.248756	0.082494
		0.003564	0.025512	0.001046	-0.151875	0.206708	0.053532	-0.158807	0.236292	0.064980

**Table 2:** Failure time of electrical appliances in terms of 1000 s cycles.

34	59	61	69	80	123	142	165	210	381
479	556	574	839	917	969	991	1064	1088	1091
1270	1275	1355	1397	1477	1578	1649	1702	1893	1932
2161	2292	2326	2337	2628	2785	2811	2886	2993	3122
3715	3790	3857	3912	4100	4106	4116	4315	4510	4584
5299	5583	6065	9701						

**Table 3:** The results of fitting some suitable models to the data set.

The model	The method	Estimations	K-S statistics	K-S *p* value	AIC
Model (2)	EM	${\hat{\alpha }}_{1}=0.62125,{\hat{\lambda }}_{1}=0.00011$	0.05246	0.9936	-
		${\hat{\alpha }}_{2}=1.98826,{\hat{\lambda }}_{2}=0.00021$			
		${\hat{\lambda }}_{3}=0.00012$			

Model (2)	MLE	${\hat{\alpha }}_{1}=0.62445,{\hat{\lambda }}_{1}=0.00011$	0.05359	0.9917	1049.014
		${\hat{\alpha }}_{2}=1.99617,{\hat{\lambda }}_{2}=0.00021$			
		${\hat{\lambda }}_{3}=0.00012$			

Model (20)	MLE	$\hat{\alpha }=0.00023,\hat{\lambda }=0.62891$	0.09987	0.5539	1044.176

## Data Availability

The lifetime data used to support the findings of this study are included within the article.

## Supplementary Materials

Supplementary materials The R codes used for simulation study and applications are presented in a supplementary file.
