# Supplementary material for: EM Algorithm for Estimating the Parameters of Weibull Competing Risk Model
Source: Appl Bionics Biomech. 2021 Oct 21;2021:1179856. doi: 10.1155/2021/1179856 (PMC8553452; doi:10.1155/2021/1179856)
Supplement: Supplementary materials — The R codes used for simulation study and applications are presented in a supplementary file. [file 1179856.f1.docx]

# The simulation study for MLE

# The baseline Weibull reliability function

Re<-function(a,la,x){

return(exp(-(la*x)^a))

}

# The competing risk reliability function

Rel<-function(a1,la1,a2,la2,a3,la3,x){

return(Re(a1,la1,x)*Re(a2,la2,x)*Re(a3,la3,x))

}

# The cdf of the competing risk model

cdf<-function(a1,la1,a2,la2,a3,la3,x){

1-Rel(a1,la1,a2,la2,a3,la3,x)

}

# The density function of the baseline Weibull

fi<-function(a,la,x){

if(a == 1){

return(la*exp(-la*x))

}

return(a*la^a*x^(a-1)*exp(-(la*x)^a))

}

# The density function of the competing risk

f<-function(a1,la1,a2,la2,a3,la3,x){

return(fi(a1,la1,x)*Re(a2,la2,x)*Re(a3,la3,x)+fi(a2,la2,x)*Re(a1,la1,x)*Re(a3,la3,x)+fi(a3,la3,x)*Re(a1,la1,x)*Re(a2,la2,x))

}

# Simulation of additive weibull

sim.add<-function(n, r, a1, la1, a2, la2, la3){

X<- matrix(0,n,r)

for(j in 1:r){

x1<-rweibull(n,a1,1/la1)

x2<-rweibull(n,a2,1/la2)

x3<-rexp(n,la3)

X[ ,j]<-pmin(x1,x2,x3)

}

list(X=X)

}

#TEST

sim1<-sim.add(2, 50, 0.8,0.01,1.3, 0.09, 0.02)

mean(sim1$X[,2])

sim1$X[,2]

#End of simulation

# MLE - First likelihood function

add.lik<- function(pars, x){

a1<-pars[1]

la1<-pars[2]

a2<-pars[3]

la2<-pars[4]

la3<-pars[5]

if(a1<0 | la1<0 | a2<0 | la2<0 | la3<0){return(100000000)}

l<- sum(log(a1*la1^a1*x^(a1 -1) + a2*la2^a2*x^(a2 -1) + la3)) - sum((la1*x)^a1+(la2*x)^a2+(la3*x))

return(-l)

}

# Optimization for MLE

optim.add<-function(te.0, x){

opt<-optim(te.0, function(par, y){add.lik(par, y)}, y=x)

est.te<-opt$par

list(est.te = est.te)

}

#TEST optim

sim1<-sim.add(60, 2, 0.8,0.01,1.3, 0.01, 0.02)

xx<-sim1$X[,2]

optim.add(c(0.7,0.021,1.13, 0.007, 0.03),xx)

#OKY

# /////////////////////////

# Simulating and computing the reults

sim.res.mle<-function(n,r,a1,la1,a2,la2,la3){

nem<-sim.add(n,r,a1,la1,a2,la2,la3)

te.real<-c(a1,la1,a2,la2,la3)

a1.hat <-c()

la1.hat<-c()

a2.hat <-c()

la2.hat<-c()

la3.hat<-c()

for(i in 1:r){

te.0<-te.real * runif(5,1.12,1.13)

o<-optim.add(te.0,nem$X[,i])

a1.hat[i]<-o$est.te[1]

la1.hat[i]<-o$est.te[2]

a2.hat[i]<-o$est.te[3]

la2.hat[i]<-o$est.te[4]

la3.hat[i]<-o$est.te[5]

}

a1.b<-mean(a1.hat-a1)

a1.ab<-mean(abs(a1.hat-a1))

a1.mse<-mean((a1.hat-a1)^2)

la1.b<-mean(la1.hat-la1)

la1.ab<-mean(abs(la1.hat-la1))

la1.mse<-mean((la1.hat-la1)^2)

a2.b<-mean(a2.hat-a2)

a2.ab<-mean(abs(a2.hat-a2))

a2.mse<-mean((a2.hat-a2)^2)

la2.b<-mean(la2.hat-la2)

la2.ab<-mean(abs(la2.hat-la2))

la2.mse<-mean((la2.hat-la2)^2)

la3.b<-mean(la3.hat-la3)

la3.ab<-mean(abs(la3.hat-la3))

la3.mse<-mean((la3.hat-la3)^2)

list(a1.b=a1.b, a1.ab=a1.ab, a1.mse=a1.mse, la1.b=la1.b, la1.ab=la1.ab, la1.mse=la1.mse,

a2.b=a2.b, a2.ab=a2.ab, a2.mse=a2.mse,

la2.b=la2.b, la2.ab=la2.ab, la2.mse=la2.mse,

la3.b=la3.b, la3.ab=la3.ab, la3.mse=la3.mse)

}

# Running

# sim.res.mle(50, 500, 0.8, 0.01, 1.3, 0.02, 0.04)

# sim.res.mle(100, 500, 0.8, 0.01, 1.3, 0.02, 0.04)

# sim.res.mle(50, 500, 1.1, 0.1, 0.9, 0.2, 0.3)

sim.res.mle(100, 500, 1.1, 0.1, 0.9, 0.2, 0.3)

# End of the simulation study for MLE

# Simulation study for LSE

# The empirical reliability function

rel.emp<-function(data, x){

d<-sort(data)

n<-length(d)

count<-0

for(i in 1:n){

if(d[i]>x){count<-count +1}

}

return(count/n)

}

# Example

rel.emp(c(2,3,3,3,4,5,6,77),3.5)

# For a vector data, it returns the

# empirical reliability in all elemets of vector

rel.e.v<-function(data){

rel.v<-c()

n<-length(data)

for(i in 1:n){

rel.v[i]<-rel.emp(data,data[i])

}

return(rel.v)

}

sq.e<-function(pars, data){

a1<-pars[1]

la1<-pars[2]

a2<-pars[3]

la2<-pars[4]

la3<-pars[5]

if(a1<0|la1<0|a2<0|la2<0|la3<0) {return(10000000)}

si <- rel.e.v(data) - exp(-(la1*data)^a1-(la2*data)^a2-(la3*data))

s2<-sum(si^2)

return(s2)

}

# Example

sq.e(c(2,3,4,2,3,6,9,9,5,4,3,3,4,5,6,77), c(0.9,0.3,1.2,0.5,1))

# End of Example

optim.sq<-function(te.0, x){

opt<-optim(te.0, function(par, y){sq.e(par, y)}, y=x)

est.te<-opt$par

list(est.te = est.te)

}

#TEST optim

sim1<-sim.add(60, 2, 0.8,0.01,1.3, 0.01, 0.02)

xx<-sim1$X[,2]

optim.sq(c(0.7,0.021,1.13, 0.007, 0.03),xx)

#OKY

# /////////////////////////

sim.res.ls<-function(n,r,a1,la1,a2,la2,la3){

nem<-sim.add(n,r,a1,la1,a2,la2,la3)

te.real<-c(a1,la1,a2,la2,la3)

a1.hat <-c()

la1.hat<-c()

a2.hat <-c()

la2.hat<-c()

la3.hat<-c()

for(i in 1:r){

te.0<-te.real * runif(5,1.02,1.03)

o<-optim.sq(te.0,nem$X[,i])

a1.hat[i]<-o$est.te[1]

la1.hat[i]<-o$est.te[2]

a2.hat[i]<-o$est.te[3]

la2.hat[i]<-o$est.te[4]

la3.hat[i]<-o$est.te[5]

}

a1.b<-mean(a1.hat-a1)

a1.ab<-mean(abs(a1.hat-a1))

a1.mse<-mean((a1.hat-a1)^2)

la1.b<-mean(la1.hat-la1)

la1.ab<-mean(abs(la1.hat-la1))

la1.mse<-mean((la1.hat-la1)^2)

a2.b<-mean(a2.hat-a2)

a2.ab<-mean(abs(a2.hat-a2))

a2.mse<-mean((a2.hat-a2)^2)

la2.b<-mean(la2.hat-la2)

la2.ab<-mean(abs(la2.hat-la2))

la2.mse<-mean((la2.hat-la2)^2)

la3.b<-mean(la3.hat-la3)

la3.ab<-mean(abs(la3.hat-la3))

la3.mse<-mean((la3.hat-la3)^2)

list(a1.b=a1.b, a1.ab=a1.ab, a1.mse=a1.mse, la1.b=la1.b, la1.ab=la1.ab, la1.mse=la1.mse,

a2.b=a2.b, a2.ab=a2.ab, a2.mse=a2.mse,

la2.b=la2.b, la2.ab=la2.ab, la2.mse=la2.mse,

la3.b=la3.b, la3.ab=la3.ab, la3.mse=la3.mse)

}

# Running

# sim.res.ls(50, 500, 0.8, 0.01, 1.3, 0.02, 0.04)

# sim.res.ls(50, 500, 1.1, 0.1, 0.9, 0.2, 0.3)

# End of simulation study for LSE

# Simulation study for EM algorithm

# x: a vector of data points

# te.t: a vector of the 5 parameters

pi.t<-function(te.t, x){

a1.t<- te.t[1]

la1.t<-te.t[2]

a2.t<- te.t[3]

la2.t<-te.t[4]

a3.t<- 1

la3.t<-te.t[5]

p1.t<-c()

p2.t<-c()

n<-length(x)

for(i in 1:n){

p1.t[i]<- fi(a1.t,la1.t,x[i])*Re(a2.t,la2.t,x[i])*Re(a3.t,la3.t,x[i])/(fi(a1.t,la1.t,x[i])*Re(a2.t,la2.t,x[i])*Re(a3.t,la3.t,x[i])

+fi(a2.t,la2.t,x[i])*Re(a1.t,la1.t,x[i])*Re(a3.t,la3.t,x[i]) + fi(a3.t,la3.t,x[i])*Re(a1.t,la1.t,x[i])*Re(a2.t,la2.t,x[i]))

p2.t[i]<- fi(a2.t,la2.t,x[i])*Re(a1.t,la1.t,x[i])*Re(a3.t,la3.t,x[i])/(fi(a1.t,la1.t,x[i])*Re(a2.t,la2.t,x[i])*Re(a3.t,la3.t,x[i])

+fi(a2.t,la2.t,x[i])*Re(a1.t,la1.t,x[i])*Re(a3.t,la3.t,x[i]) + fi(a3.t,la3.t,x[i])*Re(a1.t,la1.t,x[i])*Re(a2.t,la2.t,x[i]))

}

p3.t = 1- p1.t - p2.t

list(p1.t = p1.t, p2.t = p2.t, p3.t = p3.t)

}

Q1<-function(te.t, a,la,x){

pt1<-pi.t(te.t,x)$p1.t

q1 <- sum(pt1*(log(a)+a*log(la)+(a-1)*log(x)))-sum((la*x)^a)

return(-q1)

}

Q2<-function(te.t, a,la,x){

pt2<-pi.t(te.t,x)$p2.t

q2 <- sum(pt2*(log(a)+a*log(la)+(a-1)*log(x)))-sum((la*x)^a)

return(-q2)

}

# m: The iterations of the em procedure

em.proc<-function(te.0, x, m){

te.t<-te.0

te.tt<-c()

for(j in 1:m){

q1<-function(pars){

a<-pars[1]

la<-pars[2]

if(a<0 | la<0){ return(10000000000)}

Q1(te.t,a,la,x)

}

q2<-function(pars2){

a<-pars2[1]

la<-pars2[2]

if(a<0 | la<0){ return(10000000000)}

Q2(te.t,a,la,x)

}

a1.0 <- te.t[1]

la1.0<- te.t[2]

a2.0<- te.t[3]

la2.0<- te.t[4]

opt1<-optim(c(a1.0,la1.0),q1)

te.tt[1]<- opt1$par[1]

te.tt[2]<- opt1$par[2]

opt2<-optim(c(a2.0,la2.0),q2)

te.tt[3]<- opt2$par[1]

te.tt[4]<- opt2$par[2]

p3t<-pi.t(te.t,x)$p3.t

te.tt[5]<- sum(p3t)/sum(x)

te.t<-te.tt

}

return(te.tt)

}

# /////////////////////////

sim.res.em<-function(n,r,a1,la1,a2,la2,la3){

nem<-sim.add(n,r,a1,la1,a2,la2,la3)

te.real<-c(a1,la1,a2,la2,la3)

a1.hat <-c()

la1.hat<-c()

a2.hat <-c()

la2.hat<-c()

la3.hat<-c()

for(i in 1:r){

te.0<-te.real * runif(5,1.02,1.03)

o<-em.proc(te.0,nem$X[,i], 3)

a1.hat[i]<-o[1]

la1.hat[i]<-o[2]

a2.hat[i]<-o[3]

la2.hat[i]<-o[4]

la3.hat[i]<-o[5]

}

a1.b<-mean(a1.hat-a1)

a1.ab<-mean(abs(a1.hat-a1))

a1.mse<-mean((a1.hat-a1)^2)

la1.b<-mean(la1.hat-la1)

la1.ab<-mean(abs(la1.hat-la1))

la1.mse<-mean((la1.hat-la1)^2)

a2.b<-mean(a2.hat-a2)

a2.ab<-mean(abs(a2.hat-a2))

a2.mse<-mean((a2.hat-a2)^2)

la2.b<-mean(la2.hat-la2)

la2.ab<-mean(abs(la2.hat-la2))

la2.mse<-mean((la2.hat-la2)^2)

la3.b<-mean(la3.hat-la3)

la3.ab<-mean(abs(la3.hat-la3))

la3.mse<-mean((la3.hat-la3)^2)

list(a1.b=a1.b, a1.ab=a1.ab, a1.mse=a1.mse, la1.b=la1.b, la1.ab=la1.ab, la1.mse=la1.mse,

a2.b=a2.b, a2.ab=a2.ab, a2.mse=a2.mse,

la2.b=la2.b, la2.ab=la2.ab, la2.mse=la2.mse,

la3.b=la3.b, la3.ab=la3.ab, la3.mse=la3.mse)

}

# Running

# sim.res.em(50, 500, 0.8, 0.01, 1.3, 0.02, 0.04)

sim.res.em(100, 500, 1.1, 0.1, 0.9, 0.2, 0.3)

# End of simulation study for EM algorithm

# Application

x1<-c(0014,0034,0059,0061,0069,0080,0123,0142,0165,0210,0381, 0464,0479,0556,0574,

0839,0917,0969,0991,1064,1088,1091,1174,1270,1275,1355,1397,1477,1578,1649,1702,1893,

1932,2001,2161,2292,2326,2337,2628,2785,2811,2886,2993,3122, 3248,3715,3790,3857,

3912,4100,4106,4116,4315,4510,4584, 5267,5299,5583,6065,9701)

# TTT plot

ttt.p<-function(x){

x<-sort(x)

n<-length(x)

D<-c()

S<-c()

U<-c()

horiz<-c()

D[1]<- n*x[1]

S[1]<-D[1]

horiz[1]<-1/n

for(j in 2:n){

D[j]<-(n-j+1)*(x[j]-x[j-1])

S[j]<-S[j-1]+D[j]

horiz[j]<-j/n

}

U <- S/S[n]

list(horiz=horiz, D=D, S=S, U=U)

}

t.r<-ttt.p(x1)

plot(t.r$horiz, t.r$U)

segments(0,0,1,1)

# End of TTT plot

# Estimating parameters by EM algorithm

em.hat<-em.proc(c(0.9,0.0005,1.18,0.0005,0.0005),x1,24)

cdf0<-function(x){

cdf(em.hat[1],em.hat[2],em.hat[3],em.hat[4],1,em.hat[5],x)

}

# The K-S test for EM

ks.test(x1,cdf0)

em.hat

ml.hat<-optim.add(em.hat,x1)

ml.hat

#ml.hat2<-optim.add(c(0.9,0.0005,1.18,0.0005,0.0005),x1)

#ml.hat2

cdf1<-function(x){

cdf(ml.hat$est.te[1],ml.hat$est.te[2],ml.hat$est.te[3],ml.hat$est.te[4],1,ml.hat$est.te[5],x)

}

ks.test(x1,cdf1)

# Drawing plots

x<-seq(0, 9000, 1)

plot(x, h(em.hat[1],em.hat[2],em.hat[3],em.hat[4],1,em.hat[5], x),

type = "l", col='#b807f5', lwd=3,ylim=c(0,0.003), xlab="x", ylab="The hazard rate function")

plot(x, Rel(em.hat[1],em.hat[2],em.hat[3],em.hat[4],1,em.hat[5], x),

type = "l", col='#b807f5', lwd=3,ylim=c(0,1), xlab="x", ylab="The reliabilty function")

#//////// ////////////////////

# Drawing plots for reduced model

# The reduced model fitted by Shafaei et al.

# The MLE for reduced model

add.lik0<- function(pars, x){

a1<-pars[1]

la1<-pars[2]

if(a1<0 | la1<0 ){return(100000000)}

a2<-1/a1

la2<-la1

l<- sum(log(a1*la1^a1*x^(a1 -1) + a2*la2^a2*x^(a2 -1))) - sum((la1*x)^a1+(la2*x)^a2)

return(-l)

}

optim.add<-function(te.0, x){

opt<-optim(te.0, function(par, y){add.lik0(par, y)}, y=x)

est.te<-opt$par

list(est.te = est.te, value=opt$value)

}

#optim(em.hat, function(par, y){add.lik(par, y)}, y=x1)

ml.hat0<-optim.add(c(0.6017109, 0.00008700729),x1)

ml.hat0

cdf.reduced.1<-function(x){

cdf.reduced(ml.hat0$est.te[1],ml.hat0$est.te[2],1/ml.hat0$est.te[1],ml.hat0$est.te[2],x)

}

ks.test(x1,cdf.r)

#Empirical cdf

ecdf<-function(data,x){

i<-ifelse(data<=x, 1,0)

return(sum(i)/length(i))

}

ecdf.x1<-c()

for(i in 1:length(x1)){

ecdf.x1[i]<-ecdf(x1, x1[i])

}

t1<-seq(0,8000,1)

cdf.reduced.x1<-c()

for(i in 1:length(t1)){

cdf.reduced.x1[i]<-cdf.reduced.1(t1[i])

}

cdf.em.x1<-c()

for(i in 1:length(t1)){

cdf.em.x1[i]<-cdf0(t1[i])

}

cdf.ml.x1<-c()

for(i in 1:length(t1)){

cdf.ml.x1[i]<-cdf1(t1[i])

}

plot(x1,ecdf.x1, type='s',col='black', lwd=3,ylim=c(0,1),

xlab="Number of cycles to failure", ylab="Distribution function", cex.lab=1)

points(t1, cdf.reduced.x1, type='l',lty=2, col= 'red', lwd=3)

points(t1, cdf.ml.x1, type='l',lty=3, col= 'orange', lwd=3)

points(t1, cdf.em.x1, type='l',lty=4, col= 'green', lwd=3)

legend("bottomright",

legend = c(expression(paste("The empirical distribution function")),

expression(paste("The reduced model with two parameters (MLE)")),

expression(paste("The five parameters model (MLE)")),

expression(paste("The five parameters model (EM)"))

),

col = c("black", "red", "orange", "green") ,

lty=c(1,2,3,4,5),

lwd=c(3,3,3,3,3),

box.lty=0,

cex = 0.9)

savePlot(file="D:\\ex1-cdf", type="pdf")

# ///////////////////// MRL ///////////////////////

mrl<-function(a1,la1,a2,la2,a3,la3,x){

up<- integrate(function(t){Rel(a1,la1,a2,la2,a3,la3,t)}, x,1000000)$value

down<- Rel(a1,la1,a2,la2,a3,la3,x)

return(up/down)

}

integrate(function(x){x}, 0,1)$value+1

mrl(em.hat[1],em.hat[2],em.hat[3],em.hat[4],1,em.hat[5],x[20])

mrl.v<-c()

for(i in 1:length(x)){

mrl.v[i]<-mrl(em.hat[1],em.hat[2],em.hat[3],em.hat[4],1,em.hat[5],x[i])

}

plot(x, mrl.v,type = "l",

col='#b807f5', lwd=3,ylim=c(800,2350), xlab="x", ylab="The MRL and median residual life")

# \\\\\\\\\\\\\\\\\\\\\\\\\\\\\\\\\\\\\\\\\\\\\\\\\\\\\\\\\\\\\\\\ The QRL - Med-RL plot

inverse = function (f, lower = 0, upper = Inf) {

function (a1,la1,a2,la2,a3,la3,y) uniroot((function (x) f(a1,la1,a2,la2,a3,la3,x) - y), lower = lower, upper = upper)[1]

}

R.inv = inverse(Rel, lower=0, upper=100000)

#TEST

R.inv(em.hat[1],em.hat[2],em.hat[3],em.hat[4],1,em.hat[5],0.75)

#END OKY

qrl <- function(a1,la1,a2,la2,a3,la3,p,x){

R.inv(a1,la1,a2,la2,a3,la3,(1-p)*Rel(a1,la1,a2,la2,a3,la3,x))$root -x

}

qrl<-Vectorize(qrl)

x<-seq(0, 7000, 1)

points(x, qrl(em.hat[1],em.hat[2],em.hat[3],em.hat[4],1,em.hat[5],0.5, x), type = "l", col='red', lwd=3,ylim=c(800,2000), xlab="x", ylab="Median residual life function")

legend("topright",

legend = c(expression(paste("The MRL function")),

expression(paste("The median residual life function"))

),

col = c("#b807f5", "red") ,

lty=c(1,1),

lwd=c(3,3,3,3,3),

box.lty=0

)

segments(163.6,0,163.6,2258.228, lty=2, col="#b807f5", lwd=1)

segments(213.5,0,213.5,1823.804, lty=2, col="red", lwd=1)

segments(163.6,2258.228,1000,2258.228, lty=2, col="#b807f5", lwd=1)

segments(213.5,1823.804,1000,1823.804, lty=2, col="red", lwd=1)

text(1800,2258,cex =0.75, col= "#b807f5", "(163.6,2258.228)")

text(1800,1823,cex =0.75, col= "red", "(213.5,1823.804)")

savePlot(file="D:\\ex1-mrl-medrl", type="pdf")

tb<-seq(0,1000,0.1)

length(tb)

mrl.v.max<-c()

med.v.max<-c()

for(i in 1:length(tb)){

mrl.v.max[i]<-mrl(em.hat[1],em.hat[2],em.hat[3],em.hat[4],1,em.hat[5],tb[i])

med.v.max[i]<-qrl(em.hat[1],em.hat[2],em.hat[3],em.hat[4],1,em.hat[5],0.5, tb[i])

}

max(mrl.v.max) # 2258.228

max(med.v.max) # 1823.804

which.max(mrl.v.max)

which.max(med.v.max)

tb[which.max(mrl.v.max)] # 163.6

tb[which.max(med.v.max)] # 213.5
